# How FocA facilitates fermentation and respiration of formate by *Escherichia coli*

**DOI:** 10.1128/jb.00502-24

**Published:** 2025-01-27

**Authors:** R. Gary Sawers

**Affiliations:** 1 Institute for Microbiology, Martin Luther University Halle-Wittenberg 9176 , Halle (Saale), Saxony-Anhalt, Germany; Geisel School of Medicine at Dartmouth, Hanover, New Hampshire, USA

**Keywords:** anaerobic respiration, fermentation, formate dehydrogenase, formate hydrogenlyase complex, formate translocation

## Abstract

Formic acid is an important source of reductant and energy for many microorganisms. Formate is also produced as a fermentation product, e.g., by enterobacteria like *Escherichia coli*. As such, formic acid shares many features in common with dihydrogen, explaining perhaps why their metabolism and physiology show considerable overlap. At physiological pH, formic acid is mainly present as the dissociated formate anion and therefore cannot diffuse freely across the cytoplasmic membrane. Specific and bidirectional translocation of formate across the cytoplasmic membrane is, however, achieved in *E. coli* by the homopentameric membrane protein, FocA. Formic acid translocation from the cytoplasm into the periplasm (efflux) serves to maintain a near-neutral cytosolic pH and to deliver formate to the periplasmically-oriented respiratory formate dehydrogenases, Fdh-N and Fdh-O. These enzymes oxidize formate, with the electrons being used to reduce nitrate, oxygen, or other acceptors. In the absence of exogenous electron acceptors, formate is re-imported into the cytoplasm by FocA, where it is sensed by the transcriptional regulator FhlA, resulting in induction of the formate regulon. The genes and operons of the formate regulon encode enzymes necessary to assemble the formate hydrogenlyase complex, which disproportionates formic acid into H_2_ and CO_2_. Combined, these mechanisms of dealing with formate help to maintain cellular pH homeostasis and are suggested to maintain the proton gradient during growth and in stationary phase cells. This review highlights our current understanding of how formate metabolism helps balance cellular pH, how it responds to the redox status, and how it helps conserve energy.

## INTRODUCTION

The ability of certain enterobacteria, such as *Escherichia coli*, to disproportionate formic acid into the gaseous products H_2_ and CO_2_ has been known for well over a century (1, 2). *E. coli* is, however, able to respire with formate, using it as an electron donor: this capability was discovered toward the middle of the last century (3), despite the responsible enzyme activities having been measured in crude cell extracts some 20 years earlier (4). Although the respiratory oxidation of formate by *E. coli* is best characterized when nitrate is used as a terminal electron acceptor (5), the bacterium is also able to couple the oxidation of the anion to the reduction of oxygen (3, 6).

Meanwhile, it is clear that *E. coli* encodes three formate dehydrogenases (Fdh), whose synthesis is differentially regulated in response to the prevailing growth conditions (7, 8): Fdh-N is present at a low level under anoxic conditions, but synthesis is increased significantly during growth in the presence nitrate; Fdh-O is made under oxic as well as anoxic conditions; and synthesis of Fdh-H is induced in response to formate under fermentative growth conditions when both O_2_ and nitrate are absent (9). All three Fdhs have an active site that includes a selenocysteine residue and a *bis-*molybdopterin guanine dinucleotide cofactor, details of which have been reviewed (10–12). Despite all three oxidoreductases belonging to the dimethyl sulfoxide reductase family of molybdoenzymes, Fdh-H differs phylogenetically compared to the related respiratory Fdh-N and Fdh-O enzymes, which share a high degree of structural as well as mechanistic similarities (13–16).

**Fig 1:**
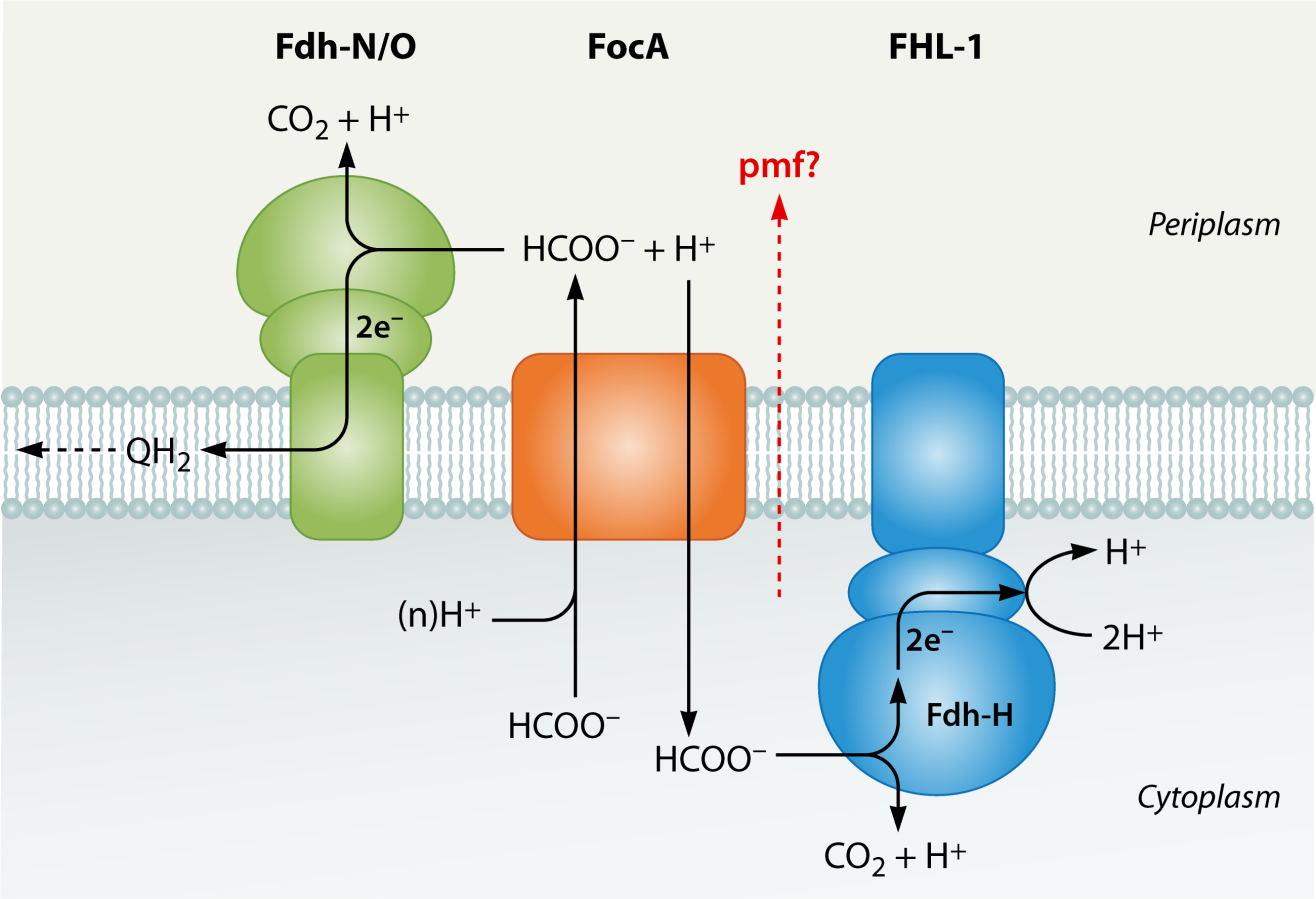
Schematic overview of formate metabolism in *E. coli*. Formate can be either disproportionated by the formate hydrogenlyase complex (FHL-1) intracellularly or oxidized by the respiratory formate dehydrogenases, Fdh-O or Fdh-N, located in the periplasm. The redox equivalents released upon formate oxidation are used to reduce the quinone pool (QH_2_, quinol). FocA translocates formate together with minimally one proton (n ≥ 1H^+^) from the cytoplasm to the periplasm, while uptake of formate is driven by the proton gradient, but the H^+^ is suggested to be reused within FocA to translocate the next and subsequent formate anions into the cytoplasm (19). The red dotted arrow through the membrane with the question mark signifies that the combined activities of FocA and FHL-1 may contribute to energy conservation.

All three Fdh enzymes are membrane-associated, but unlike both respiratory Fdhs, which have their respective active site located on the periplasmic side of the membrane, Fdh-H has its active site located in the cytoplasm, as the enzyme is a component of the multisubunit formate hydrogenlyase (FHL) complex (Fig. 1; 8). This means that the side of the membrane on which formate happens to be located, and whether an exogenous electron acceptor is available, both have a major impact on how formate is further metabolized. Consequently, this also significantly affects the physiology of the bacterium. While the Fdh-N enzyme uses menaquinone as an electron acceptor (14), recent evidence strongly suggests that the Fdh-O enzyme transfers electrons to ubiquinone (17), perhaps explaining why *E. coli* maintains two very similar enzymes that help conserve energy using Mitchell’s “redox loop” mechanism of proton motive force (*pmf*) generation (18).

In contrast, Fdh-H transfers electrons to a protein within the FHL complex (20, 21) and not to a quinone. Indeed, recent findings indicate that Fdh-H likely functions as a flexible module and is a subunit of two different FHL complexes (FHL-1 and FHL-2), and perhaps also other multiprotein complexes (22, 23), supplying protons and electrons for H_2_ generation or ion movement across the membrane. As will be discussed below, these FHL complexes are considered to contribute either directly or indirectly to energy conservation (22, 24), which is also in accord with Fdh-H being located on the inner leaflet of the cytoplasmic membrane.

What these three Fdhs have in common is their substrate, formate, which, along with acetyl-coenzyme A (acetyl-CoA), is the product of CoA-dependent cleavage of pyruvate by the glycyl-radical enzyme, pyruvate formate-lyase (PflB) (Fig. 1; 25, 26). As well as being a fermentation product, formate is also a valuable electron donor for biosynthesis and energy conservation, it is essential for anaerobic DNA synthesis (27), and it has an important role in purine biosynthesis (28). Formate is also a potential source of CO_2_ for heterotrophic carboxylation reactions. Moreover, with a p*K*_a_ of 3.75, formate is 10-fold more acidic than acetate, having the consequence that with careful regulation of its oxidation or disproportionation, it can be used by the bacterium to tune pH homeostasis (29, 30). For this modulation of pH to be controlled effectively, formate, or formic acid, must be translocated bidirectionally across the membrane. Bidirectional formate translocation is controlled by the pentameric membrane channel/secondary transporter, FocA, which consequently plays a central role in determining how, when, and where formate is metabolized (30). Thus, this current review will focus on how the micro-aerobic and anaerobic physiology of *E. coli* is governed in response to this chemically simple organic acid.

## WHEN IS FORMATE MADE?

Despite being a highly O_2_-sensitive glycyl-radical enzyme, PflB is nonetheless synthesized at a significant level during aerobic growth; however, under oxic conditions, PflB is enzymically inactive (26). While PflB is held in this inactive state during aerobic growth, pyruvate is oxidatively cleaved by the pyridine nucleotide- and CoA-dependent pyruvate dehydrogenase (Pdh) complex (31). PflB is inactive because under oxic conditions the PflB-activating enzyme, PflA, lacks one of the substrates, reduced ferredoxin (Fd_red_), which is required along with *S*-adenosyl methionine to generate and introduce the organic radical that is essential for PflB enzyme activity (25, 26). As such, regulation of PflB enzyme activity is responsive to the cellular redox status. Thus, when O_2_ levels decrease, Fd_red_ levels increase and PflB activation occurs rapidly to allow pyruvate cleavage and acetyl-CoA generation to continue. At the same time, expression of the *focA-pflB* operon is induced 10- to 12-fold through the action of the O_2_-responsive iron-sulfur-containing fumarate and nitrate reduction (FNR) transcriptional regulator and the redox-responsive ArcAB (aerobic respiration control) two-component system (32, 33), which is activated by sensing an increase in the quinol:quinone ratio of the respiratory chain (34). Enhanced PflB enzyme synthesis coupled with a high catalytic turnover rate (35) means that pyruvate is immediately homolytically cleaved to acetyl-CoA and formate.

When nitrate is present as an electron acceptor, expression of the *focA-pflB* operon is decreased (36) but nevertheless, sufficient enzyme is still made and the redox potential of the cell is such that it is maintained in an active state. This is indicated by the fact that either the Pdh complex or PflB can cleave pyruvate during nitrate respiration (37), accounting for formate synthesis and provision as an electron donor for nitrate reduction (38, 39).

## REGULATION OF FDH-N AND FDH-O SYNTHESIS

Genetic analysis of the *fdnGHI* operon, encoding the structural components of the nitrate-inducible Fdh-N enzyme, has greatly facilitated the investigation of the regulation of its synthesis (40). Construction of gene and operon fusions between *fdnG* and the *lacZ* reporter allowed dissection of the regulatory mechanisms behind the well-known nitrate-dependent stimulation of formate dehydrogenase enzyme activity (41–43). Redox control of *fdnGHI* expression is mediated by the FNR regulator (41), while the presence of nitrate is sensed by the NarXL two-component system (44). Together, FNR and the phosphorylated NarL response regulator ensure that the *fdnGHI* operon is expressed maximally only in the absence of oxygen and when nitrate is available (42). Such dual hierarchical control of Fdh-N synthesis guarantees that the enzyme will only be made in high amounts in the absence of O_2_ and when nitrate is present. Moreover, the essentially identical regulatory pattern is exhibited for the expression of the *narGHJI* operon, encoding the respiratory nitrate reductase, NarGHI, which completes the energy-conserving formate-nitrate respiratory pathway (15, 45). Nevertheless, even in the absence of nitrate, low-level synthesis of both Fdh-N and NarGHI is maintained under fermentation conditions to allow a rapid switch to respiratory formate oxidation should the exogenous electron acceptor become available (46).

The long-forgotten “formate oxidase” activity originally identified by Pinsent (3) was revitalized from the late 1980s onward through the analysis of the Fdh-O enzyme activity and identification of the *fdoGHI* operon encoding the enzyme (6, 15). Transcriptional reporter studies revealed that in contrast to the *fdn* operon, the expression of *fdo* is not responsive to changes in redox status or the presence of nitrate (15). Despite a minor increase in operon expression in the presence of O_2_, synthesis of the Fdh-O enzyme is essentially constitutive, although a more recent study suggests that enzyme synthesis might be increased in the late stationary phase (47). Together, the regulation and synthesis of Fdh-O and Fdh-N ensure that the cell is primed for energy-conserving *pmf* generation if formate is available as a respiratory electron donor.

## FORMATE LEVELS DETERMINE THE SYNTHESIS OF FDH-H

**Fig 2:**
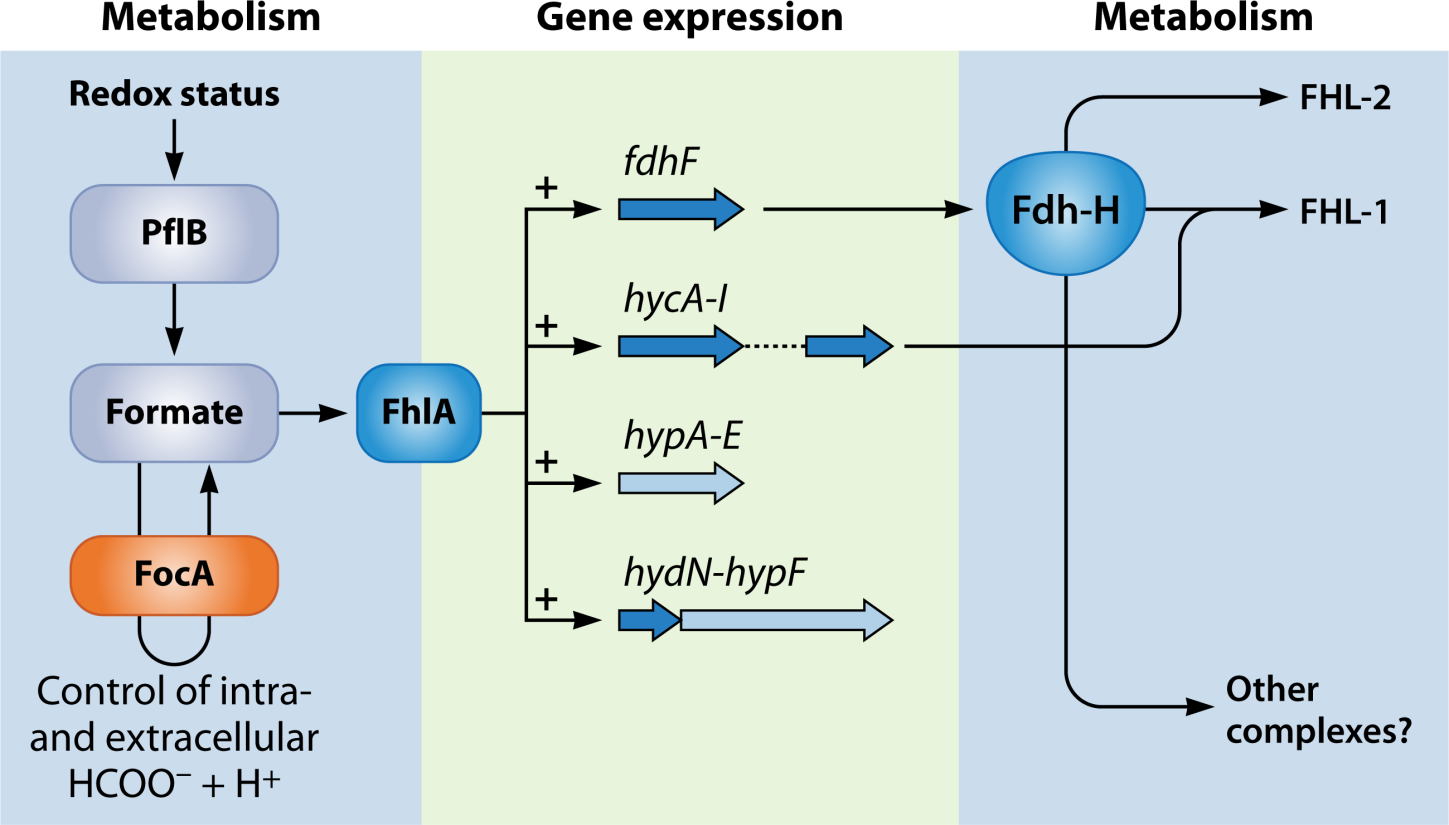
Overview of the formate regulon and how formate is generated and metabolized in the cytoplasm. Formate is generated by PflB (left). The activity of PflB is responsive to the cellular redox status (lack of O_2_ and the availability of reduced ferredoxin; 25) and translocation of formate between the cytoplasm and the periplasm is controlled by FocA. Cytoplasmic accumulation of formate activates the transcriptional regulator FhlA, which induces the expression of the genes and operons represented schematically in the center of the figure. The products of these genes then form part or all of the enzyme complexes indicated on the right side of the figure. Hyf indicates the subunits of hydrogenase 4 (53), which combine with Fdh-H to form the FHL-2 complex. The *hyc* operon (54) encodes the subunits of the FHL-1 complex, which also functions with Fdh-H. It is possible that Fdh-H also delivers electrons to other, as yet unknown, enzyme complexes (see 23).

The gene encoding the Fdh-H enzyme that is associated with both the FHL-1 and the FHL-2 complex is part of the formate-FhlA regulon (Fig. 2) (9). FhlA is an orphan two-component transcriptional regulator that responds directly to intracellular formate levels and coordinately induces the transcription of all of the genes whose products are necessary for the synthesis of an active FHL-1 complex (48, 49). The approximately 5 mM concentration of formate necessary to bind half of the FhlA molecules in the cell (50, 51) sets the threshold for FHL-1 synthesis. Consequently, when formate is drained from the cytoplasm by the activity of the periplasmic respiratory Fdh-O and -N enzymes, this automatically prevents expression of FhlA-regulated genes and operons and curtails synthesis and activity of FHL-1 (8, 9). Indeed, this can be observed readily in a very simple experiment whereby, when mM concentrations of either O_2_ or nitrate are added to an H_2_-evolving culture of *E. coli*, gas production stops instantaneously; the inhibitory effect of nitrate on H_2_ evolution was described already in 1901 (2). Thus, the hierarchical control of formate metabolism is imposed simply by the presence of either O_2_ or nitrate (9). Even accumulation of low intracellular levels of fumarate, which derives ultimately from carboxylation of phosphoenolpyruvate during anaerobic growth, diverts formate to the respiratory Fdhs, and this fumarate is used as an electron acceptor to oxidize the quinol pool (52).

## FDH-H IS MODULAR AND CAN COUPLE WITH MORE THAN ONE ELECTRON-TRANSFER COMPLEX

It was something of a surprise when the gene encoding Fdh-H, *fdhF*, was discovered to be located on a quite distinct part of the *E. coli* genome compared with the *hyc* operon, which encodes the rest of the structural components of the formate-inducible FHL-1 complex (Fig. 2) (13, 54). It has been known for nigh on 35 years that the expression of both *fdhF* and *hyc* is controlled by the transcription factor FhlA (49), which, in turn, is responsive to intracellular formate levels (50, 55). Another component suggested to be important in electron transfer in the FHL-1 complex is the iron-sulfur-containing protein HydN (23, 56). Yet, the *hydN* gene is co-transcribed with the *hypF* gene (Fig. 2), encoding a carbamoyltransferase involved in [NiFe]-cofactor biosynthesis (57). The *hydN-hypF* operon is also separately located on the chromosome from the *hyc* operon, but nevertheless, expression is also enhanced by formate-FhlA as it is part of the formate regulon (9). Together, these findings suggest that the Fdh-H protein, and possibly also HydN (23), may be modular in function and can associate with other electron-transfer complexes. Indeed, it has recently been shown that Fdh-H confers upon the FHL-2 complex the ability in stationary phase cells to use formate as an electron donor to evolve H_2_ and possibly also to generate an ion gradient (22). Meanwhile, using bacterial two-hybrid screening, HydN has been shown to interact with Fdh-H (23) and more recently HydN has been identified as a structural component of the FHL-2 complex in the enterobacterium *Trabulsiella guamensis* (M. Hardelt, R.G. Sawers, C. Pinske, unpublished data).

## MOVING FORMATE BETWEEN THE CYTOPLASM AND PERIPLASM VIA FOCA

Rapid, bidirectional translocation of formate between the cytoplasm and the periplasm is a requirement to make this substrate readily available for the three Fdhs, depending on the prevailing growth conditions. For example, when O_2_ or nitrate becomes available, rapid translocation of formate to the periplasm for Fdh-O and -N, respectively, would maximize energy conservation and at the same time prevent undesirable acidification of the cytoplasm. Equally, when exogenous electron acceptors are no longer available, a means of efficient translocation of formate back into the cytoplasm would help offset acidification of the periplasm and allow disproportionation of formic acid to neutral gaseous products (29). One means by which energy-efficient and speedy formate translocation can be achieved is by means of a channel. Thirty years ago, such a channel for formate translocation was identified and termed FocA (58). Meanwhile, numerous genes encoding phylogenetically related membrane proteins that exhibit specificity for different monovalent anions and their cognate acids have been identified. These are collectively referred to as the formate-nitrite transporter (FNT) family (30, 59).

Structural analyses of FocA and other FNTs have revealed that the proteins all share a conserved pentameric structure resembling the aqua-glyceroporins (19, 60–63). Each, approximately 31 kDa, protomer of the pentamer has a hydrophobic pore containing two highly conserved amino acid residues, T91 and H209 (based on *E. coli* FocA residue numbering). These residues are located centrally within the pore and can interact to form a hydrogen bond (62, 63). Mutagenesis studies using *E. coli* FocA have demonstrated not only that both of these residues are important for bidirectional translocation of formate, but also that the mechanisms of efflux and uptake differ (64). These findings indicate that pores of this family of proteins do not act as a simple open-or-closed channel (65).

Other studies have also shown that PflB interacts with the cytoplasmically oriented *N*-terminal helix of FocA (66, 67). Interaction with PflB is essential for *in vivo* bidirectional formate translocation to occur. Moreover, the removal of the *N*-terminal helix of FocA severely disrupts FocA’s ability to translocate formate *in vivo* (67) and this correlates with an inability of T91 and H209 to form a hydrogen bond *in vitro* (60). It is presumed, therefore, that the interaction of PflB with FocA not only gates the channel, but also confers specificity for the formate, delivers the anion directly into the channel pore for translocation, and ensures the formation of the hydrogen bond between T91 and H209, which opens the pore (30, 68).

## EFFLUX OF FORMIC ACID BY FOCA

Based on the hydrophobicity of the pore, it has been posited that a charged formate anion would be unable to pass through the pore to the periplasm and that only the neutral acid would be able to be translocated (69). Recent studies have indeed shown that formate and a proton are translocated out of the cytoplasm (53); the stoichiometry of HCOO^−^:H^+^ is unresolved. Nevertheless, the T91 and H209 residues are involved in this efflux of formic acid, and it is possible that they influence sterically how rapidly the molecule passes through the pore (64, 70). This presumption is borne out by mutagenesis studies, which reveal that an efflux-only phenotype results when H209 is exchanged for a small neutral residue, whereas exchange for a bulky aromatic residue or an amino acid with a charged side-chain severely impedes formic acid efflux (71). These phenotypes also allow the conclusion to be drawn that the protonatable histidine at position 209 is important for formate uptake (see below).

Exchange of T91 for any other residue except serine also severely hinders formic acid efflux, which indicates that, although channel-like behavior through the pore is observed in the efflux direction, a hydroxylated residue within hydrogen-bonding distance of H209 is a prerequisite to allow passage of formic acid through the pore (68, 70, 71).

Although the H209 residue is highly conserved across the thousands of members of the FNT family so far identified, a handful of FNTs have either asparagine or glutamine in this position (72). While these residues can readily hydrogen bond with the hydroxyl group of threonine, they cannot be protonated. The introduction of either of these residues at amino acid position 209 results in a FocA protein with a very efficient, efflux-only phenotype (64). As well as efficiently excreting formic acid, a strain synthesizing a FocA-H209N variant exhibits a reduced capacity to grow to high optical density by fermentation in a minimal medium, causing an early entry of the cells into the stationary phase (64). This growth phenotype appears to correlate with reduced cellular levels of ATP (C. Erdmann and R.G. Sawers, unpublished data).

## UPTAKE OF FORMATE BY FOCA IS PH-DEPENDENT AND COUPLED WITH FHL-1 ACTIVITY

Early studies on glucose fermentation by *E. coli* showed that FHL-1-dependent H_2_ production is increased at pH values below 6.5 (73). This pH dependence of H_2_ production correlates strongly with the presence of the H209 residue in FocA and the ability of the bacterium to import formate and synthesize FHL-1 (9, 30). Indeed, a mutant unable to synthesize the FHL-1 complex, either through mutation or when formic acid is efficiently excreted out of the cell so that it cannot accumulate and activate FhlA, e.g., due to synthesis of the FocA-H209N protein (63), fails to re-import formic acid once it has been excreted (74).

*E. coli* mutants lacking FocA were originally isolated based on the resistance of the mutants to the toxic chemical analog of formate, hypophosphite (58). Hypophosphite irreversibly inhibits PflB enzyme activity, causing reduced growth during fermentation (26). Because *focA* mutants are unable to import hypophosphite, they exhibit resistance to it, with the consequence that fermentative growth is improved, even in the presence of the compound (58). However, the characteristic of hypophosphite that is important is that it has a p*K*_a_ of 1.1, which means that under physiological growth conditions, it is always present as the dissociated anionic species. All amino acid-exchange variants of FocA that lack histidine at position 209 fail to import hypophosphite, indicating that protonation of the anion by the histidine residue is essential for its uptake through the pores of FocA. Furthermore, these findings demonstrate unequivocally that the mechanisms of formic acid efflux and uptake by FocA differ. The data also show that uptake of formate by FocA is proton-driven and this necessitates proton delivery to formate by the histidine residue (p*K*_a_ of the free amino acid is 6), potentially explaining the pH-dependence of formate uptake and H_2_ production (73).

Notably, residue T91 (or its substitution variant, S91) is also essential for the uptake of formate and hypophosphite by FocA, even when H209 is present. This indicates that the threonine residue is mechanistically involved in the protonation of formate by H209. A mechanism for this reaction has been suggested (68), based on an initial proposal made during the structural analysis of another FNT family member, the nitrite transporter, NirC (63). At acidic pH, the proton from the imidazolium cation is proposed to be transferred to formate, which has entered from the periplasmic side of the pore. The water-coordinated T91 residue, which is hydrogen-bonded to H209 and is located lower within the pore and closer to the cytoplasmic side of the membrane (19, 60–63), transfers its hydroxyl proton to the imidazole group, reforming the strong imidazolium cation. The resulting alkoxide anion of T91 then recaptures the proton from formic acid once it passes the most hydrophobic part of the pore, releasing formate into the cytoplasm (63, 68). Recapture of the proton from formic acid would be consistent with an energy-conserving mechanism, especially if the coupled formate disproportionation reaction catalyzed by the FHL-1 complex uses a scalar cytoplasmic proton together with formate to generate H_2_ (68).

## WHAT FUNCTIONAL RELEVANCE DOES FOCA HAVE FOR THE FDHS AND THE *E. COLI* CELL?

Current data suggest that FocA serves minimally two roles in catabolic formate metabolism. During growth, the first, and likely major, role, functioning together with the FHL-1 complex, is the maintenance of near-neutral intracellular pH during fermentation. This is necessary because of the acidity of formic acid, which is similar to that of lactic acid. Rapid efflux of formic acid to the periplasmic side of the membrane by the channel-like function of FocA alleviates acidification of the cytoplasm and at the same time offers formate as a substrate to the respiratory Fdh enzymes, allowing reduction of any available electron acceptor, i.e., O_2_, nitrate, or fumarate, should either of these acceptors become available.

The other role of FocA is that of a less rapid secondary transport system for the uptake of formate, either directly from the cellular environment or of the formate that was originally translocated from the cytoplasm to the periplasm. Both of these possibilities have been shown to occur (29, 58, 67, 74). Again, this facilitates pH homeostasis by dissipating the acid as neutral gases and concomitantly increases periplasmic pH (29, 68). Such a role in pH homeostasis would be dependent on a functional FHL-1 system to blow off H_2_, and this has been demonstrated (58, 74), but it could be argued that this function could be equally fulfilled by a periplasmic Fdh-H. However, because the FHL-1 complex is located at the inner leaflet of the cytoplasmic membrane, this argues strongly in favor of formate uptake helping to maintain the *pmf*, which is especially important during energy-limited survival in the stationary phase. This correlates with the growth phase in which the FHL-1 complex is most active (29, 30, 73). If efflux of formate, along with one or more protons, and subsequent re-uptake of only the anion by FocA occurs (Fig. 1), then coupling this process with disproportionation of the formate anion to H_2_ and CO_2_ (consuming a further scalar proton from the cytoplasm) by FHL-1 would formally contribute to *pmf* generation, as recently proposed (68). If the released H_2_ can be oxidized by the periplasmically-localized respiratory hydrogenases 1 and 2 (29, 68, 75), this would further enhance the energy-conserving capacity of this system.

Re-import of formate by FocA, or its paralog FocB encoded as part of the *hyf* operon (76), in stationary-phase cells and coupling of FdhH with the FHL-2 complex might be an alternative means of contributing to energy conservation. The FHL-2 complex is proposed to be a progenitor of complex 1 of the respiratory chain and accumulating evidence suggests that it might also be energy-conserving in function (24, 54, 77–79).

The importance of FocA in balancing intracellular formate concentration has been shown (70), as has the dramatic consequences of disrupting this balance by introducing an amino acid variant of FocA (e.g., FocA-H209N), which efficiently exports formic acid and causes premature entry into the stationary phase (64). This growth phenotype can be partially rectified by cultivation of the strain in a rich medium, suggesting that the early entry into the stationary phase could be due either to a reduction in ATP levels causing growth restriction, to an imbalance in intracellular pH, or increased carbon flux through glycolysis. Notably, and in agreement with the proposed role of FocA in facilitating the translocation of formate/formic acid to the side of the membrane where it is immediately required, initial findings indicate that when nitrate is added to the growth medium of an *E. coli* strain synthesizing the FocA-H209N variant, the growth rate of the strain is initially increased significantly due to delivery of formate to the nitrite-inducible Fdh-N-NAR respiratory pathway (C. Erdmann and R.G. Sawers, unpublished data).

## CONCLUSIONS AND THE BROADER PERSPECTIVE

The importance of the balanced production and further metabolism of formate during oxygen-restricted growth of *E. coli* is becoming more apparent. The interwoven nature of formate and hydrogen, CO_2_, and proton metabolisms has been crucial during evolution and continues to have a central role in the cellular metabolism of modern microorganisms. Analysis of how formic acid/formate is distributed across the cytoplasmic membrane reveals potentially simple and ancient links in the homeostatic control of cellular energy, carbon, redox, and pH to maintain ion gradients across the membrane. This is exemplified in the broader context by the thermophilic archaeon *Thermococcus onnurineus*, which can grow by formate-driven H_2_ production (80), long considered inconceivable, and by the well-established growth of methanogenic archaea that use H_2_ and formate for energy and carbon, respectively (81, 82). The use of formate as a substrate for the Wood-Ljungdahl pathway in the acetogenic bacteria, *Thermoanaerobacter kivui* and *Acetobacterium woodii* (83, 84), similarly points to an ancient origin of formate-driven or -directed processes, with the former species synthesizing an H_2_-dependent CO_2_ reductase for formate generation (84, 85). Similarly, formate is produced by certain marine *Vibrio* species as a means of off-loading reducing equivalents (86), as in *E. coli*. Despite having been first described nearly 40 years ago (87) more recent studies on the use of formic acid as an electron shuttle in microbial communities are also beginning to reveal the importance of formate in driving syntrophic processes in anaerobic habitats (88, 89), including in the mammalian intestine (90). All of these examples highlight the importance of formate metabolism for the growth and survival of microorganisms in anoxic and micro-oxic habitats.

How widespread formate as a respiratory energy source is in natural habitats is also becoming clearer with the demonstration of its use as an electron donor by *Campylobacter jejuni*, which has a periplasmic Fdh enzyme that allows coupling of formate oxidation with microaerobic O_2_ reduction (91), again in analogy to the role of Fdh-O in *E. coli*. Similarly, a recent report suggests the presence of an O_2_-insensitive tungsten-containing formate dehydrogenase in *Desulfovibrio vulgaris*, which apparently can couple with O_2_ reduction (92). Moreover, the requirement for radical chemistry in carbon-bond cleavage underscores how evolutionarily ancient these interlinked processes are, especially in the generation of formate. The FocA channel-cum-secondary transporter is a membrane protein with the capability of controlling the distribution of formate and protons across the cytoplasmic membrane using simple and energy-efficient mechanisms and is also found in thermophilic archaea, as well as marine *Vibrio* species that perform a mixed-acid-type fermentation (80, 86). Coupling the balanced distribution of formate and protons with intracellular disproportionation of the acid and extracellular formate oxidation is delivering new insights into how a simple mechanism can be used to help maintain ion gradients and pH homeostasis under highly challenging and energy-limiting conditions.

## References

[1] Hoppe-Seyler F. 1876. Ueber die processe der Gährungen und ihre Beziehung zum leben der organismen. Pflüger Arch 12:1–17. doi:10.1007/BF01640188

[2] Pakes WCC, Jollyman WH. 1901. XL.—The bacterial decomposition of formic acid into carbon dioxide and hydrogen. J Chem Soc Trans 79:386–391. doi:10.1039/CT9017900386

[3] Pinsent J. 1954. The need for selenite and molybdate in the formation of formic dehydrogenase by members of the coli-aerogenes group of bacteria. Biochem J 57:10–16. doi:10.1042/bj0570010 13159942 PMC1269698

[4] Yudkin J. 1933. The dehydrogenases of Bacterium coli. Biochem J 17:1849–1858. 10.1042/bj0271849 PMC1253109 16745309

[5] Ruiz-Herrera J, Showe MK, DeMoss JA. 1969. Nitrate reductase complex of Escherichia coli K-12: isolation and characterization of mutants unable to reduce nitrate. J Bacteriol 97:1291–1297. doi:10.1128/jb.97.3.1291-1297.1969 4887509 PMC249846

[6] Sawers G, Heider J, Zehelein E, Böck A. 1991. Expression and operon structure of the sel genes of Escherichia coli and identification of a third selenium-containing formate dehydrogenase isoenzyme. J Bacteriol 173:4983–4993. doi:10.1128/jb.173.16.4983-4993.1991 1650339 PMC208187

[7] Sawers G. 1994. The hydrogenases and formate dehydrogenases of Escherichia coli. Antonie Van Leeuwenhoek 66:57–88. doi:10.1007/BF00871633 7747941

[8] Pinske C, Sawers RG. 2016. Anaerobic formate and hydrogen metabolism. EcoSal Plus 7. doi:10.1128/ecosalplus.ESP-0011-2016 PMC11575713 27735784

[9] Rossmann R, Sawers G, Böck A. 1991. Mechanism of regulation of the formate-hydrogenlyase pathway by oxygen, nitrate, and pH: definition of the formate regulon. Mol Microbiol 5:2807–2814. doi:10.1111/j.1365-2958.1991.tb01989.x 1779767

[10] Böck A, Forchhammer K, Heider J, Baron C. 1991. Selenoprotein synthesis: an expansion of the genetic code. Trends Biochem Sci 16:463–467. doi:10.1016/0968-0004(91)90180-4 1838215

[11] Jormakka M, Byrne B, Iwata S. 2003. Formate dehydrogenase--a versatile enzyme in changing environments. Curr Opin Struct Biol 13:418–423. doi:10.1016/s0959-440x(03)00098-8 12948771

[12] Leimkühler S. 2020. The biosynthesis of the molybdenum cofactors in Escherichia coli. Environ Microbiol 22:2007–2026. doi:10.1111/1462-2920.15003 32239579

[13] Zinoni F, Birkmann A, Stadtman TC, Böck A. 1986. Nucleotide sequence and expression of the selenocysteine-containing polypeptide of formate dehydrogenase (formate-hydrogen-lyase-linked) from Escherichia coli. Proc Natl Acad Sci U S A 83:4650–4654. doi:10.1073/pnas.83.13.4650 2941757 PMC323799

[14] Jormakka M, Törnroth S, Byrne B, Iwata S. 2002. Molecular basis of proton motive force generation: structure of formate dehydrogenase-N. Science 295:1863–1868. doi:10.1126/science.1068186 11884747

[15] Abaibou H, Pommier J, Benoit S, Giordano G, Mandrand-Berthelot M-A. 1995. Expression and characterization of the Escherichia coli fdo locus and a possible physiological role for aerobic formate dehydrogenase. J Bacteriol 177:7141–7149. doi:10.1128/jb.177.24.7141-7149.1995 8522521 PMC177593

[16] Soboh B, Pinske C, Kuhns M, Waclawek M, Ihling C, Trchounian K, Trchounian A, Sinz A, Sawers G. 2011. The respiratory molybdo-selenoprotein formate dehydrogenases of Escherichia coli have hydrogen: benzyl viologen oxidoreductase activity. BMC Microbiol 11:173. doi:10.1186/1471-2180-11-173 21806784 PMC3160892

[17] Kashyap DR, Kowalczyk DA, Shan Y, Yang C-K, Gupta D, Dziarski R. 2020. Formate dehydrogenase, ubiquinone, and cytochrome bd-I are required for peptidoglycan recognition protein-induced oxidative stress and killing in Escherichia coli. Sci Rep 10:1993. doi:10.1038/s41598-020-58302-1 32029761 PMC7005000

[18] Mitchell P. 1979. Keilin’s respiratory chain concept and its chemiosmotic consequences. Science 206:1148–1159. doi:10.1126/science.388618 388618

[19] Lü W, Du J, Wacker T, Gerbig-Smentek E, Andrade SLA, Einsle O. 2011. pH-dependent gating in a FocA formate channel. Science 332:352–354. doi:10.1126/science.1199098 21493860

[20] McDowall JS, Murphy BJ, Haumann M, Palmer T, Armstrong FA, Sargent F. 2014. Bacterial formate hydrogenlyase complex. Proc Natl Acad Sci U S A 111:E3948–E3956. doi:10.1073/pnas.1407927111 25157147 PMC4183296

[21] Steinhilper R, Höff G, Heider J, Murphy BJ. 2022. Structure of the membrane-bound formate hydrogenlyase complex from Escherichia coli. Nat Commun 13:5395. doi:10.1038/s41467-022-32831-x 36104349 PMC9474812

[22] Mirzoyan S, Romero-Pareja PM, Coello MD, Trchounian A, Trchounian K. 2017. Evidence for hydrogenase-4 catalyzed biohydrogen production in Escherichia coli. Int J Hydrogen Energy 42:21697–21703. doi:10.1016/j.ijhydene.2017.07.126

[23] Pinske C. 2018. The ferredoxin-like proteins HydN and YsaA enhance redox dye-linked activity of the formate dehydrogenase H component of the formate hydrogenlyase complex. Front Microbiol 9:1238. doi:10.3389/fmicb.2018.01238 29942290 PMC6004506

[24] Pinske C. 2019. Bioenergetic aspects of archaeal and bacterial hydrogen metabolism. Adv Microb Physiol 74:487–514. doi:10.1016/bs.ampbs.2019.02.005 31126536

[25] Backman LRF, Funk MA, Dawson CD, Drennan CL. 2017. New tricks for the glycyl radical enzyme family. Crit Rev Biochem Mol Biol 52:674–695. doi:10.1080/10409238.2017.1373741 28901199 PMC5911432

[26] Knappe J, Sawers G. 1990. A radical-chemical route to acetyl-CoA: the anaerobically induced pyruvate formate-lyase system of Escherichia coli. FEMS Microbiol Rev 6:383–398. doi:10.1111/j.1574-6968.1990.tb04108.x 2248795

[27] Jordan A, Reichard P. 1998. Ribonucleotide reductases. Annu Rev Biochem 67:71–98. doi:10.1146/annurev.biochem.67.1.71 9759483

[28] Marolewski A, Smith JM, Benkovic SJ. 1994. Cloning and characterization of a new purine biosynthetic enzyme: a non-folate glycinamide ribonucleotide transformylase from E. coli. Biochemistry 33:2531–2537. doi:10.1021/bi00175a023 8117714

[29] Metcalfe GD, Sargent F, Hippler M. 2022. Hydrogen production in the presence of oxygen by Escherichia coli K-12. Microbiology (Reading) 168:3. doi:10.1099/mic.0.001167 PMC9558352 35343886

[30] Kammel M, Pinske C, Sawers RG. 2022. FocA and its central role in fine-tuning pH homeostasis of enterobacterial formate metabolism. Microbiology (Reading) 168:10. doi:10.1099/mic.0.001253 36197793

[31] Patel MS, Nemeria NS, Furey W, Jordan F. 2014. The pyruvate dehydrogenase complexes: structure-based function and regulation. J Biol Chem 289:16615–16623. doi:10.1074/jbc.R114.563148 24798336 PMC4059105

[32] Sawers G, Böck A. 1988. Anaerobic regulation of pyruvate formate-lyase from Escherichia coli K-12. J Bacteriol 170:5330–5336. doi:10.1128/jb.170.11.5330-5336.1988 3053657 PMC211609

[33] Sawers G, Suppmann B. 1992. Anaerobic induction of pyruvate formate-lyase gene expression is mediated by the ArcA and FNR proteins. J Bacteriol 174:3474–3478. doi:10.1128/jb.174.11.3474-3478.1992 1592804 PMC206030

[34] Georgellis D, Kwon O, Lin EC. 2001. Quinones as the redox signal for the arc two-component system of bacteria. Science 292:2314–2316. doi:10.1126/science.1059361 11423658

[35] Knappe J, Elbert S, Frey M, Wagner AF. 1993. Pyruvate formate-lyase mechanism involving the protein-based glycyl radical. Biochem Soc Trans 21 (Pt 3):731–734. doi:10.1042/bst0210731 8135930

[36] Kaiser M, Sawers G. 1995. Nitrate repression of the Escherichia coli pfl operon is mediated by the dual sensors NarQ and NarX and the dual regulators NarL and NarP. J Bacteriol 177:3647–3655. doi:10.1128/jb.177.13.3647-3655.1995 7601827 PMC177079

[37] Kaiser M, Sawers G. 1994. Pyruvate formate-lyase is not essential for nitrate respiration by Escherichia coli. FEMS Microbiol Lett 117:163–168. doi:10.1111/j.1574-6968.1994.tb06759.x 8181719

[38] Ruiz-Herrera J, DeMoss JA. 1969. Nitrate reductase complex of Escherichia coli K-12: participation of specific formate dehydrogenase and cytochrome b1 components in nitrate reduction. J Bacteriol 99:720–729. doi:10.1128/jb.99.3.720-729.1969 4905536 PMC250087

[39] Enoch HG, Lester RL. 1975. The purification and properties of formate dehydrogenase and nitrate reductase from Escherichia coli. J Biol Chem 250:6693–6705. 1099093

[40] Berg BL, Stewart V. 1990. Structural genes for nitrate-inducible formate dehydrogenase in Escherichia coli K-12. Genetics 125:691–702. doi:10.1093/genetics/125.4.691 2168848 PMC1204095

[41] Li J, Stewart V. 1992. Localization of upstream sequence elements required for nitrate and anaerobic induction of fdn (formate dehydrogenase-N) operon expression in Escherichia coli K-12. J Bacteriol 174:4935–4942. doi:10.1128/jb.174.15.4935-4942.1992 1629153 PMC206306

[42] Darwin AJ, Li J, Stewart V. 1996. Analysis of nitrate regulatory protein NarL-binding sites in the fdnG and narG operon control regions of Escherichia coli K-12. Mol Microbiol 20:621–632. doi:10.1046/j.1365-2958.1996.5491074.x 8736541

[43] Wang H, Gunsalus RP. 2003. Coordinate regulation of the Escherichia coli formate dehydrogenase fdnGHI and fdhF genes in response to nitrate, nitrite, and formate: roles for NarL and NarP. J Bacteriol 185:5076–5085. doi:10.1128/JB.185.17.5076-5085.2003 12923080 PMC180993

[44] Rabin RS, Stewart V. 1993. Dual response regulators (NarL and NarP) interact with dual sensors (NarX and NarQ) to control nitrate- and nitrite-regulated gene expression in Escherichia coli K-12. J Bacteriol 175:3259–3268. doi:10.1128/jb.175.11.3259-3268.1993 8501030 PMC204722

[45] Walker MS, DeMoss JA. 1991. Promoter sequence requirements for Fnr-dependent activation of transcription of the narGHJI operon. Mol Microbiol 5:353–360. doi:10.1111/j.1365-2958.1991.tb02116.x 2041473

[46] Cole JA, Richardson DJ. 2008. Respiration of nitrate and nitrite. EcoSal Plus 3. doi:10.1128/ecosal.3.2.5 26443731

[47] Iwadate Y, Funabasama N, Kato J-I. 2017. Involvement of formate dehydrogenases in stationary phase oxidative stress tolerance in Escherichia coli. FEMS Microbiol Lett 364:fnx193. doi:10.1093/femsle/fnx193 29044403

[48] Sankar P, Lee JH, Shanmugam KT. 1988. Gene-product relationships of fhlA and fdv genes of Escherichia coli. J Bacteriol 170:5440–5445. doi:10.1128/jb.170.12.5440-5445.1988 3056900 PMC211635

[49] Schlensog V, Böck A. 1990. Identification and sequence analysis of the gene encoding the transcriptional activator of the formate hydrogenlyase system of Escherichia coli. Mol Microbiol 4:1319–1327. doi:10.1111/j.1365-2958.1990.tb00711.x 2280686

[50] Hopper S, Böck A. 1995. Effector-mediated stimulation of ATPase activity by the sigma 54-dependent transcriptional activator FHLA from Escherichia coli. J Bacteriol 177:2798–2803. doi:10.1128/jb.177.10.2798-2803.1995 7751289 PMC176951

[51] Fardan AAA, Koestler BJ. 2024. FhlA is a formate binding protein. bioRxiv:2024.07.24.604796. doi:10.1101/2024.07.24.604796

[52] Clark DP. 1989. The fermentation pathways of Escherichia coli. FEMS Microbiol Rev 5:223–234. doi:10.1016/0168-6445(89)90033-8 2698228

[53] Vanyan L, Kammel M, Sawers RG, Trchounian K. 2024. Evidence for bidirectional formic acid translocation in vivo via the Escherichia coli formate channel FocA. Arch Biochem Biophys 752:109877. doi:10.1016/j.abb.2023.109877 38159898

[54] Böhm R, Sauter M, Böck A. 1990. Nucleotide sequence and expression of an operon in Escherichia coli coding for formate hydrogenlyase components. Mol Microbiol 4:231–243. doi:10.1111/j.1365-2958.1990.tb00590.x 2187144

[55] Hopper S, Babst M, Schlensog V, Fischer HM, Hennecke H, Böck A. 1994. Regulated expression in vitro of genes coding for formate hydrogenlyase components of Escherichia coli. J Biol Chem 269:19597–19604. 8034728

[56] Maier T, Binder U, Böck A. 1996. Analysis of the hydA locus of Escherichia coli: two genes (hydN and hypF) involved in formate and hydrogen metabolism. Arch Microbiol 165:333–341. doi:10.1007/s002030050335 8661925

[57] Paschos A, Bauer A, Zimmermann A, Zehelein E, Böck A. 2002. HypF, a carbamoyl phosphate-converting enzyme involved in [NiFe] hydrogenase maturation. J Biol Chem 277:49945–49951. doi:10.1074/jbc.M204601200 12377778

[58] Suppmann B, Sawers G. 1994. Isolation and characterization of hypophosphite--resistant mutants of Escherichia coli: identification of the FocA protein, encoded by the pfl operon, as a putative formate transporter. Mol Microbiol 11:965–982. doi:10.1111/j.1365-2958.1994.tb00375.x 8022272

[59] Saier MH Jr, Eng BH, Fard S, Garg J, Haggerty DA, Hutchinson WJ, Jack DL, Lai EC, Liu HJ, Nusinew DP, Omar AM, Pao SS, Paulsen IT, Quan JA, Sliwinski M, Tseng TT, Wachi S, Young GB. 1999. Phylogenetic characterization of novel transport protein families revealed by genome analyses. Biochim Biophys Acta 1422:1–56. doi:10.1016/s0304-4157(98)00023-9 10082980

[60] Wang Y, Huang Y, Wang J, Cheng C, Huang W, Lu P, Xu Y-N, Wang P, Yan N, Shi Y. 2009. Structure of the formate transporter FocA reveals a pentameric aquaporin-like channel. Nature New Biol 462:467–472. doi:10.1038/nature08610 19940917

[61] Waight AB, Love J, Wang D-N. 2010. Structure and mechanism of a pentameric formate channel. Nat Struct Mol Biol 17:31–37. doi:10.1038/nsmb.1740 20010838 PMC3613427

[62] Czyzewski BK, Wang DN. 2012. Identification and characterization of a bacterial hydrosulphide ion channel. Nature New Biol 483:494–497. doi:10.1038/nature10881 PMC3711795 22407320

[63] Lü W, Schwarzer NJ, Du J, Gerbig-Smentek E, Andrade SLA, Einsle O. 2012. Structural and functional characterization of the nitrite channel NirC from Salmonella typhimurium. Proc Natl Acad Sci U S A 109:18395–18400. doi:10.1073/pnas.1210793109 23090993 PMC3494889

[64] Kammel M, Trebbin O, Pinske C, Sawers RG. 2022. A single amino acid exchange converts FocA into A unidirectional efflux channel for formate. Microbiology (Reading) 168:001132. doi:10.1099/mic.0.001132 35084298 PMC8914244

[65] Armstrong CM. 2015. Packaging life: the origin of ion-selective channels. Biophys J 109:173–177. doi:10.1016/j.bpj.2015.06.012 26200853 PMC4621617

[66] Doberenz C, Zorn M, Falke D, Nannemann D, Hunger D, Beyer L, Ihling CH, Meiler J, Sinz A, Sawers RG. 2014. Pyruvate formate-lyase interacts directly with the formate channel FocA to regulate formate translocation. J Mol Biol 426:2827–2839. doi:10.1016/j.jmb.2014.05.023 24887098 PMC5560055

[67] Kammel M, Hunger D, Sawers RG. 2021. The soluble cytoplasmic N-terminal domain of the FocA channel gates bidirectional formate translocation. Mol Microbiol 115:758–773. doi:10.1111/mmi.14641 33169422

[68] Kammel M, Erdmann C, Sawers RG. 2024. The formate-hydrogen axis and its impact on the physiology of enterobacterial fermentation. Adv Microb Physiol 84:51–82. doi:10.1016/bs.ampbs.2024.02.002 38821634

[69] Atkovska K, Hub JS. 2017. Energetics and mechanism of anion permeation across formate-nitrite transporters. Sci Rep 7:12027. doi:10.1038/s41598-017-11437-0 28931899 PMC5607303

[70] Kammel M, Sawers RG. 2022. The FocA channel functions to maintain intracellular formate homeostasis during Escherichia coli fermentation. Microbiology (Reading) 168:4. doi:10.1099/mic.0.001168 35377837

[71] Kammel M, Trebbin O, Sawers RG. 2022. Interplay between the conserved pore residues Thr-91 and his-209 controls formate translocation through the FocA channel. Microb Physiol 32:95–107. doi:10.1159/000524454 35390794

[72] Helmstetter F, Arnold P, Höger B, Petersen LM, Beitz E. 2019. Formate-nitrite transporters carrying nonprotonatable amide amino acids instead of a central histidine maintain pH-dependent transport. J Biol Chem 294:623–631. doi:10.1074/jbc.RA118.006340 30455351 PMC6333897

[73] Stephenson M, Stickland LH. 1932. Hydrogenlyases: bacterial enzymes liberating molecular hydrogen. Biochem J 26:712–724. doi:10.1042/bj0260712 16744879 PMC1260964

[74] Beyer L, Doberenz C, Falke D, Hunger D, Suppmann B, Sawers RG. 2013. Coordination of FocA and pyruvate formate-lyase synthesis in Escherichia coli demonstrates preferential translocation of formate over other mixed-acid fermentation products. J Bacteriol 195:1428–1435. doi:10.1128/JB.02166-12 23335413 PMC3624525

[75] Sawers RG. 1985. Membrane-bound hydrogenase isoenzymes of Escherichia coli. PhD Dissertation, University of Dundee, U.K.

[76] Andrews SC, Berks BC, McClay J, Ambler A, Quail MA, Golby P, Guest JR. 1997. A 12-cistron Escherichia coli operon (hyf) encoding A putative proton-translocating formate hydrogenlyase system. Microbiology (Reading) 143 (Pt 11):3633–3647. doi:10.1099/00221287-143-11-3633 9387241

[77] Finney AJ, Lowden R, Fleszar M, Albareda M, Coulthurst SJ, Sargent F. 2019. The plant pathogen Pectobacterium atrosepticum contains a functional formate hydrogenlyase-2 complex. Mol Microbiol 112:1440–1452. doi:10.1111/mmi.14370 31420965 PMC7384014

[78] Lindenstrauß U, Pinske C. 2019. Dissection of the hydrogen metabolism of the enterobacterium Trabulsiella guamensis: identification of a formate-dependent and essential formate hydrogenlyase complex exhibiting phylogenetic similarity to complex I. J Bacteriol 201:e00160-19. doi:10.1128/JB.00160-19 30962355 PMC6531613

[79] Peters K, Sargent F. 2023. Formate hydrogenlyase, formic acid translocation and hydrogen production: dynamic membrane biology during fermentation. Biochim Biophys Acta Bioenerg 1864:148919. doi:10.1016/j.bbabio.2022.148919 36152681

[80] Kim YJ, Lee HS, Kim ES, Bae SS, Lim JK, Matsumi R, Lebedinsky AV, Sokolova TG, Kozhevnikova DA, Cha S-S, Kim S-J, Kwon KK, Imanaka T, Atomi H, Bonch-Osmolovskaya EA, Lee J-H, Kang SG. 2010. Formate-driven growth coupled with H2 production. Nature New Biol 467:352–355. doi:10.1038/nature09375 20844539

[81] Ferry JG. 1999. Enzymology of one-carbon metabolism in methanogenic pathways. FEMS Microbiol Rev 23:13–38. doi:10.1111/j.1574-6976.1999.tb00390.x 10077852

[82] Holden JF, Sistu H. 2023. Formate and hydrogen in hydrothermal vents and their use by extremely thermophilic methanogens and heterotrophs. Front Microbiol 14:1093018. doi:10.3389/fmicb.2023.1093018 36950162 PMC10025317

[83] Burger Y, Schwarz FM, Müller V. 2022. Formate-driven H2 production by whole cells of Thermoanaerobacter kivui. Biotechnol Biofuels 15:48. doi:10.1186/s13068-022-02147-5 PMC9097184 35545791

[84] Moon J, Dönig J, Kramer S, Poehlein A, Daniel R, Müller V. 2021. Formate metabolism in the acetogenic bacterium Acetobacterium woodii. Environ Microbiol 23:4214–4227. doi:10.1111/1462-2920.15598 33989450

[85] Schwarz FM, Schuchmann K, Müller V. 2018. Hydrogenation of CO_2_ at ambient pressure catalyzed by a highly active thermostable biocatalyst. Biotechnol Biofuels 11:237. doi:10.1186/s13068-018-1236-3 30186365 PMC6119302

[86] Sato Y, Mino S, Thompson F, Sawabe T. 2024. Core transcriptome of hydrogen producing marine Vibrios reveals contribution of glycolysis in their efficient hydrogen production. Curr Microbiol 81:230. doi:10.1007/s00284-024-03764-z 38896159

[87] Thiele JH, Zeikus JG. 1988. Control of interspecies electron flow during anaerobic digestion: significance of formate transfer versus hydrogen transfer during syntrophic methanogenesis in flocs. Appl Environ Microbiol 54:20–29. doi:10.1128/aem.54.1.20-29.1988 16347526 PMC202391

[88] Montag D, Schink B. 2018. Formate and hydrogen as electron shuttles in terminal fermentations in an oligotrophic freshwater lake sediment. Appl Environ Microbiol 84:e01572-18. doi:10.1128/AEM.01572-18 30097443 PMC6182907

[89] Westerholm M, Calusinska M, Dolfing J. 2022. Syntrophic propionate-oxidizing bacteria in methanogenic systems. FEMS Microbiol Rev 46:1–26. doi:10.1093/femsre/fuab057 PMC8892533 34875063

[90] Trischler R, Roth J, Sorbara MT, Schlegel X, Müller V. 2022. A functional wood-Ljungdahl pathway devoid of a formate dehydrogenase in the gut acetogens Blautia wexlerae, Blautia luti and beyond. Environ Microbiol 24:3111–3123. doi:10.1111/1462-2920.16029 35466558

[91] Kassem II, Candelero-Rueda RA, Esseili KA, Rajashekara G. 2017. Formate simultaneously reduces oxidase activity and enhances respiration in Campylobacter jejuni. Sci Rep 7:40117. doi:10.1038/srep40117 28091524 PMC5238407

[92] Graham JE, Niks D, Zane GM, Gui Q, Hom K, Hille R, Wall JD, Raman CS. 2022. How a formate dehydrogenase responds to oxygen: unexpected O2 insensitivity of an enzyme harboring tungstopterin selenocysteine, and [4Fe−4S] clusters. ACS Catal 12. doi:10.1101/2022.01.18.476765

